# ERR*γ*, a Novel Biomarker, Associates with Pathoglycemia of Endometrial Cancer to Predict Myometrial Invasion

**DOI:** 10.1155/2022/5283388

**Published:** 2022-06-21

**Authors:** Yao Tong, Meimei Huang, Lili Chen, Huifang Lei, Hao Lin, Xiaodan Mao, Pengming Sun

**Affiliations:** ^1^Department of Gynecology, Fujian Maternity and Child Health Hospital College of Clinical Medicine for Obstetrics & Gynecology and Pediatrics, Fujian Medical University, Fuzhou 350001, China; ^2^Department of Gynecology, Fujian Obstetrics and Gynecology Hospital, Fuzhou 350001, China; ^3^Reproductive Center, Fujian Maternity and Child Health Hospital College of Clinical Medicine for Obstetrics & Gynecology and Pediatrics, Fujian Medical University, Fuzhou 350001, China; ^4^Laboratory of Gynecologic Oncology, Fujian Maternity and Child Health Hospital College of Clinical Medicine for Obstetrics & Gynecology and Pediatrics, Fujian Medical University, Fuzhou 350001, China; ^5^Key Laboratory of Women and Children's Critical Diseases Research, Fujian Maternity and Child Health Hospital College of Clinical Medicine for Obstetrics & Gynecology and Pediatrics, Fujian Medical University, Fuzhou 350001, China; ^6^School of Medical Technology and Engineering, Fujian Medical University, No. 88 Jiaotong Road, Fuzhou 350004, China; ^7^National Health Commission Key Laboratory of Technical Evaluation of Fertility Regulation for Non-human Primate, Jinjishan Road, Fuzhou 350005, China

## Abstract

We aim to investigate the correlation between the expression of estrogen-related receptor *γ* (ERR*γ*) and endometrial cancer (EC) progression and to evaluate the potential of ERR*γ* as a new biomarker for EC diagnosis. We analyzed the ERR*γ* expression profile and the correlation with the corresponding clinical characteristics of EC samples from The Cancer Genome Atlas (TCGA), the Clinical Proteomic Tumor Analysis Consortium (CPTAC) databases, and the International Cancer Genome Consortium (ICGC) databases. Immunohistochemical (IHC) analysis was conducted on tissue samples, and enzyme linked immunosorbent assay (ELISA) was used in serum samples to detect the levels of ERR*γ*. The diagnostic performance of ERR*γ* proteins was assessed using the receiver operating characteristic (ROC). ERR*γ* showed notably higher expression in EC tissues than in normal endometrium tissues (*P* < 0.001), which was consistent with the result of TCGA. Overexpression of ERR*γ* was significantly associated with deep myometrial invasion of EC (*P*=0.004), and fasting blood glucose (FBG) was higher in EC patients with deep myometrial invasion than in those with superficial myometrial invasion (*P*=0.040). Further analysis using ELISA showed that the serum ERR*γ* level was positively correlated with FBG (*R* = 0.355, *P* < 0.001). ERR*γ* is overexpressed in EC and may be involved in regulating glucose metabolism and promoting myometrial invasion of EC. In addition, the area under the ROC curve (AUC) for ERR*γ* was 0.834, in distinguishing EC patients from healthy individuals, presented 84.0% and 80.0% sensitivity and specificity, respectively, and serum ERR*γ* has a good diagnostic performance in distinguishing EC patients from healthy people and may be a promising noninvasive biomarker in EC.

## 1. Introduction

The incidence of endometrial cancer (EC) is increasing year by year, and it has become the first cancer of the female reproductive tract in the world [1]. Myometrial invasion in advanced EC is closely related to poor prognosis [2]. However, the mechanisms involved in the invasion and metastasis of malignant tumors are still unclear. Patients usually seek medical attention for abnormal uterine bleeding, which is found to be EC by pap-smear, curettage pathology, or hysteroscopy. Due to the lack of specific markers, it is of great significance to find reliable and valuable biomarkers for early identification and diagnosis of EC.

Abnormal glucose metabolism is a common feature often observed in patients with EC [3]. Accumulating evidence indicates that hyperglycemia is associated with poor prognosis of EC [4,5]. Recently, studies have found that the nuclear receptor estrogen-related receptor *γ* (ERR*γ*), as a metabolism-related gene, is widely involved in the regulation of several key enzymes in cell glucose metabolism, lipid metabolism, and amino acid metabolism [6]. ERR*γ* is related to abnormal gluconeogenesis, insulin resistance, and other pathological states and participates in the occurrence and development of diseases with abnormal glucose metabolism [7]. ERR*γ* is highly expressed in diabetic patients with poor blood glucose control [8] and is abnormally expressed in a variety of metabolism-related diseases, including malignant tumors, and involved in the occurrence and development of cancer [9,10].

This study aimed to investigate the relationship between ERR*γ* and EC and glucose metabolism to evaluate the role of ERR*γ* as a biomarker for the diagnosis of EC.

## 2. Materials and Methods

### 2.1. Data Acquisition and Processing

We explored mRNA expression of ERR*γ* from The Cancer Genome Atlas (TCGA) database, including 546 EC and 35 normal endometrial tissue samples. We analyzed the expression of ERR*γ* mRNA in patients with different histological subtypes and stages. Clinical Proteomic Tumor Analysis Consortium (CPTAC) analysis of the UALCAN portal (http://ualcan.path.uab.edu/analysis-prot.html.) was used to identify the protein expression level of ERR*γ* in UCEC (uterine corpus endometrial carcinoma). In addition, another dataset from the International Cancer Genome Consortium (ICGC) data portal (https://icgc.org/) was used to assess survival differences.

### 2.2. Patients

The medical records of 525 EC patients treated in Fujian Maternity and Child Health Hospital College of Clinical Medicine for Obstetrics & Gynecology and Pediatrics, Fujian Medical University from January 2012 to December 2018 were studied retrospectively. The exclusion criteria were as follows: (1) patients with a history of other malignancies; (2) patients with nonepithelial cancers of the uterus, such as carcinosarcoma; (3) patients treated with chemotherapy, radiotherapy, or hormone therapy before surgery; (4) patients missing clinical pathology data or with an unclear diagnosis; and (5) patients who did not agree for further analysis of their pathological tissue. Healthy controls were from those who had undergone curettage for other reasons such as endometrial polyps, adenomyosis, leiomyomas, hyperplasia, and hemorrhages due to congenital and acquired coagulopathies, ovarian dysfunction, and disorders of the local endometrial hemostasis mechanism with normal organ structure. Healthy controls received adequate screening and excluding for endometrial lesions or other types of malignancies and other disease during the same period. This study was approved by the Ethics Committee of Fujian Maternity and Child Health Hospital College of Clinical Medicine for Obstetrics & Gynecology and Pediatrics, Fujian Medical University (No. YCXM20-01) and performed in compliance with the Declaration of Helsinki. Informed consent was obtained from all participants included in the study.

### 2.3. Sample Preparation

**Figure 1:**
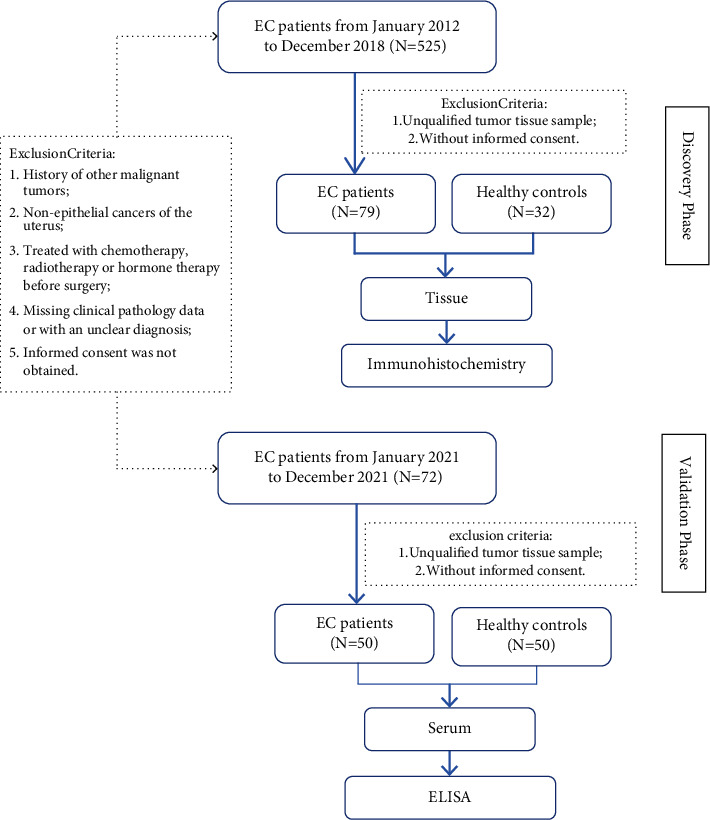
Flowchart of the study protocol.

For the discovery phase, we collected endometrial cancer tissue from 525 EC patients who had retained tissue samples during the last 7 years. Excluding unqualified samples and those without informed consent, 79 cases of endometrial carcinoma were eventually included. Among them, stage I and II patients (*n* = 63) accounted for 78.5% of the total cases and endometrioid adenocarcinoma; nonendometrioid adenocarcinoma was about 4 : 1, consistent with the epidemiological distribution [11,12]. Patients with normal endometrial pathology (*n* = 32) were collected during the same period. All tissue samples were collected during the surgery. Immunohistochemistry (IHC) was used to detect the expression of ERR*γ* in tissue chip samples. For the validation phase, serum samples from 50 EC patients from January 2021 to December 2021 were collected and paired with 50 healthy individuals. There were 41 patients in the early stage, and the stage distribution was consistent with the epidemiological characteristics (Figure 1). All serum samples were collected two days before surgery, and ERR*γ* protein was evaluated by ELISA, which were entirely separated from the discovery set samples. All methods were carried out in accordance with relevant guidelines and regulations set out below.

### 2.4. Immunohistochemistry

To examine the expression of ERR*γ* in tissue, we performed a tissue microarray constructed by Shanghai Zhuoli Biotechnology Co., Ltd (Zhuoli Biotechnology Co., Shanghai, China). Rabbit polyclonal anti-ERR*γ* (ab49129, Abcam) were used. Two pathologists independently evaluated the quantitation of immunostaining for ERR*γ*, who were blinded to patient details. The expression of ERR*γ* in tumor parenchyma was semiquantified by the immunoreactivity score (IR score) based on intensity and heterogeneity. The IR score was determined as the sum of heterogeneity and intensity. Intensity of staining was scored as 0 (negative), 1 (low), 2 (medium), and 3 (high). Area extent of staining was scored as 0 (0% stained), 1 (1–25% stained), 2 (26–50% stained), and 3 (51–100% stained). The final score was determined by multiplying the intensity scores with area extent and ranged from 0 to 9. Final scores (intensity score × percentage score) < 6 were considered as low and ≥6 were high expression.

### 2.5. Serum

*ELISA.* Blood samples were collected and centrifuged at 1500°g for 10 min. Serum samples were stored at − 70°C until the day of the analysis. The serum level of ERR*γ* was assessed by using a solid phase sandwich enzyme linked immunosorbent assay (ELISA) kit (cat. #JL48961; Jianglai Inc., Shanghai, China), following the manufacturers' protocol. The optical density of each well was then read at 450 nm using a microplate reader. Serum levels of ERR*γ* were calculated from a standard curve based on reference standards.

### 2.6. Statistical Analysis

In this study, ERR*γ* expression was compared to different groups using the chi-square or Fisher's exact test, when appropriate. Student's *t*-test was used to compare continuous variables in two groups. Pearson correlation analysis was used to evaluate the correlations between continuous variables. Survival rates were calculated using the Kaplan–Meier estimator. Receiver-operating characteristic (ROC) curves were used to evaluate the diagnostic value of ERR*γ*. The ERR*γ* cut-off value was calculated using the Youden index. All statistical analyses were performed using SPSS 22.0 (IBM, Chicago, IL, USA) and GraphPad Prism version 8.0 software (GraphPad Software, Inc., La Jolla, CA, USA). All *P* values in the statistical analysis were two-tailed. *P* < 0.05 was considered statistically significant.

## 3. Results

### 3.1. Expression of ERR*γ* in TCGA Database and the CPTAC Database

**Figure 2:**
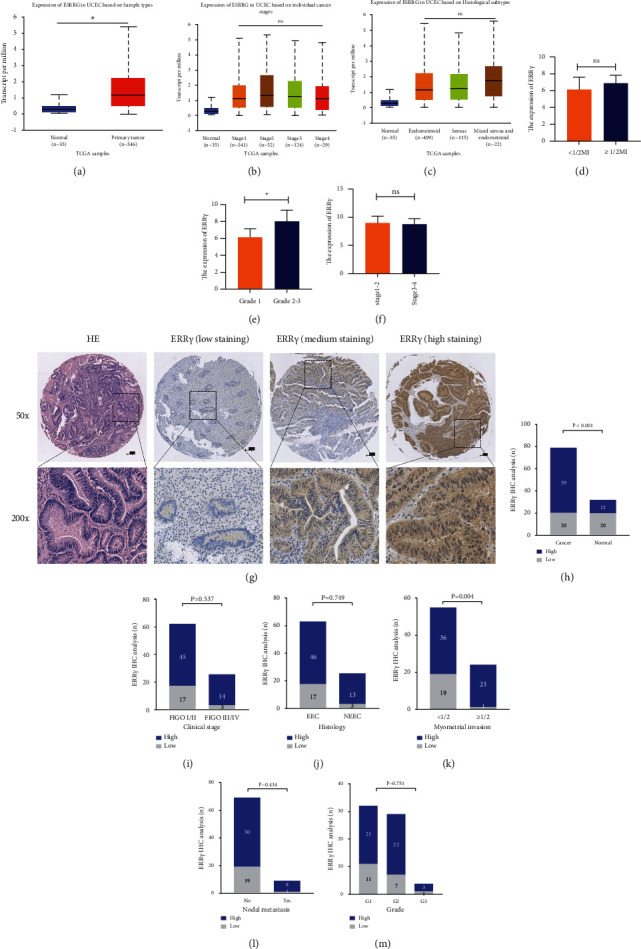
(a–c) Expression of ERR*γ* in Uterine Corpus Endometrial Carcinoma (UCEC) in TCGA database. (d–f) Expression of ERR*γ* in UCEC in the CPTAC database. (g) Immunohistochemical staining for hematoxylin/eosin (HE) and ERR*γ* on normal and tumor tissues (magnification, ×50, ×200). (h) Expression of ERR*γ* in EC and normal endometrial tissue. (i–m) Difference expression of ERR*γ* in tumor tissue with different clinical features.

As shown in Figures 2(a)–2(c), the mRNA expression profiles retrieved from TCGA revealed that the expression of ERR*γ* was significantly higher in EC than the normal sample (*P* < 0.001). There was no difference in both International Federation of Gynecology and Obstetrics (FIGO) stages and histological subtypes (*P* > 0.05). The CPTAC database showed that ERR*γ* expression was higher in the deep myometrial invasion depth group than in the superficial myometrial invasion depth group, but the difference was not statistically significant (*P* > 0.05; Figure 2(d)). The expression of ERR*γ* in G2-G3 group was higher than that in G1 group (*P* < 0.05; Figure 2(e)), while there was no statistical significance in ERR*γ* expression in different stage groups (*P* > 0.05; Figure 2(f)).

### 3.2. ERR*γ* Is Highly Expressed in EC Tissue

We analyzed the expression of ERR*γ* in 79 cases of EC tissue samples and 32 cases of healthy controls by IHC (Figure 2(g)). ERR*γ* was more highly expressed in EC tissues than in normal endometrial tissue (*P* < 0.001). Among 79 cases of EC samples, 59 samples showed high ERR*γ* expression (Figure 2(h)). In addition, we analyzed the differences in the expression of ERR*γ* in tumor tissues with different clinical features (Figures 2(i)–2(m)). We observed that high ERR*γ* expression is associated with deep myometrial invasion (*P*=0.004), and there was no statistical significance in the expression of ERR*γ* among different clinical stages, pathological types, and lymph node metastatic status groups (*P* > 0.05).

### 3.3. Overexpression of ERR*γ* Significantly Correlates with Deep Myometrial Invasion

We analyzed the correlation between the expression of ERR*γ* and immunohistochemical markers in EC tissues (Table 1). The results showed that the high expression rate of ERR*γ* in the vimentin-positive group was higher than that in the vimentin-negative group (76.8% vs. 23.2%), and Spearman correlation analysis showed a significant positive correlation between ERR*γ* and vimentin (*R* = 0.368, *P*=0.001). There was no statistical correlation between ERR*γ* and ER, PR, PTEN, P53, and Ki67 (*P* > 0.05). In addition, the expressions of vimentin and Ki67 were different in different myometrial invasion groups (*P*=0.018, *P*=0.042), and there was no significant difference in the expressions of ER, PR, PTEN, and P53 in different myometrial invasion groups (*P* > 0.05).

### 3.4. Risk Factors for Deep Myometrial Invasion in EC

**Figure 3:**
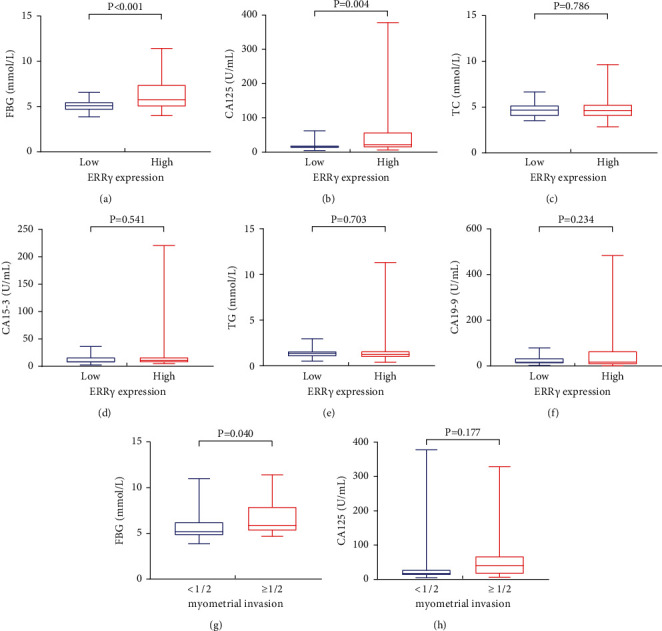
(a–f) Differences of FBG, CA125, TC, CA15-3, TG, and CA19-9 levels in different ERR*γ* expression groups of EC. (g–h) Differences of FBG and CA125 levels in different myometrial invasion depth groups of EC. FBG: fasting blood glucose; TC: cholesterol; TG: triglycerides.

According to the depth of myometrial invasion, patients with EC were divided into two groups (Table 2). Comparing the clinical information of the two groups, it was found that the age, FBG, and CA125 were higher in the deep myometrial invasion group (P˂0.001, *P*=0.001, and *P*=0.009), but there were no statistically significant differences in BMI, triglyceride, cholesterol, and other parameters between the two groups (*P* > 0.05). Age, FBG, and CA125 were risk factors for deep myometrial invasion in EC, and FBG and CA125 were still associated with the risk of deep myometrial invasion after adjustment for age (OR = 1.281, 95%Cl = 1.102-1.490, *P*=0.001 OR = 1.002, 95%Cl = 1.000–1.004, *P*=0.019; Table S1). In addition, our results showed that the high expression of ERR*γ* in EC tissue was associated with higher FBG and CA125 (P˂0.001, *P*=0.004; Figures 3(a) and 3(b)), while there was no statistical correlation with TC, TG, CA15-3, and CA19-9 (all *P* > 0.05; Figures 3(c)–3(f)). The level of FBG in the deep myometrial invasion group was higher than that in the superficial myometrial invasion group (*P*=0.040; Figure 3(g)), while the difference of CA125 in different myometrial invasion groups was not statistically significant in the 79 EC patients (*P*=0.177; Figure 3(h)).

### 3.5. Prognostic Value of the ERR*γ* Expression Level in EC Tissue

**Figure 4:**
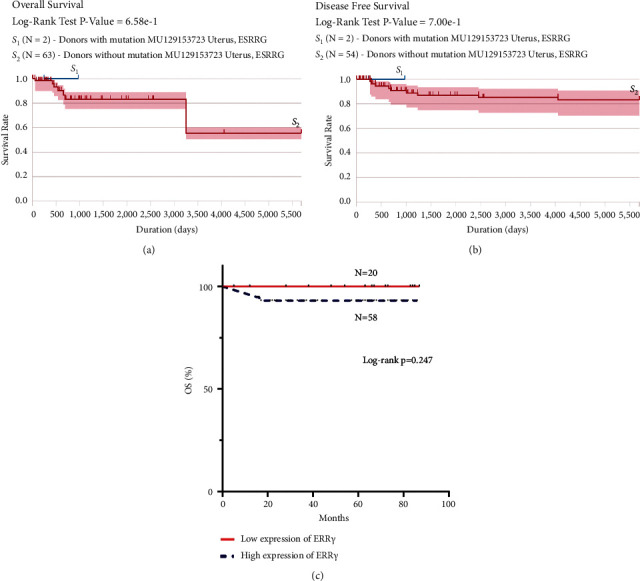
(a–b) The relationship between ERR*γ* mutation and prognosis in the ICGC database. (c) Kaplan–Meier (KM) survival curves of EC patients with different ERR*γ* expressions.

The ICGC database showed that there was no statistical difference in overall survival and disease-free survival between donors with and without ERR*γ* mutations (*P* > 0.05; Figures 4(a) and 4(b)). We followed up 79 cases of endometrial cancer with cancer tissue for nearly 7 years, including 1 case of loss to follow-up, and only 4 cases of death among 78 patients. There was no statistically significant difference in the survival rate between patients with high and low expression of ERR*γ* protein in tissues (*P* > 0.05; Figure 4(c)).

### 3.6. The Expression Levels of ERR*γ* in Serum

**Figure 5:**
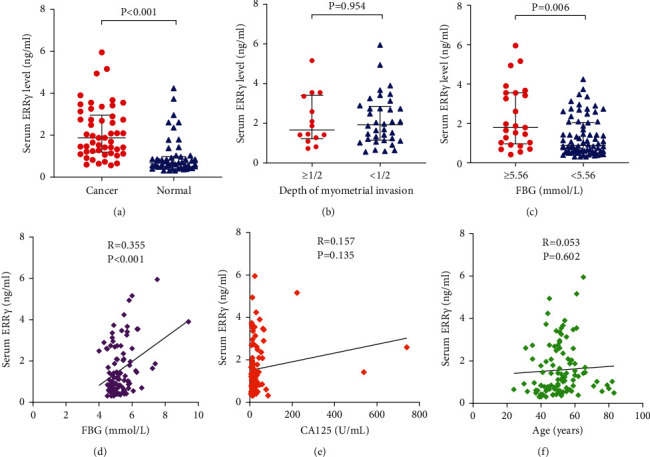
(a) Serum levels of ERR*γ* in EC patients and healthy controls. (b) Serum levels of ERR*γ* in different myometrial invasion depth groups. (c) Serum levels of ERR*γ* in different serum FBG groups. (d–f) Correlation analysis between serum ERR*γ* and other indicators in EC patients and healthy controls. FBG: fasting blood glucose.

Next, the serum ERR*γ* level was determined in the cohort of 50 EC patients and 50 control samples from healthy people by ELISA. The results showed that serum levels of ERR*γ* was significantly higher in EC patients (2.156 ± 1.254 ng/mL) than in healthy controls (0.994 ± 0.879 ng/mL, *P* < 0.001; Figure 5(a)). No significant association was shown between serum ERR*γ* levels and depth of myometrial invasion (*P*=0.954; Figure 5(b)).

### 3.7. Diagnostic Value of the Serum ERR*γ* Level in EC

**Figure 6:**
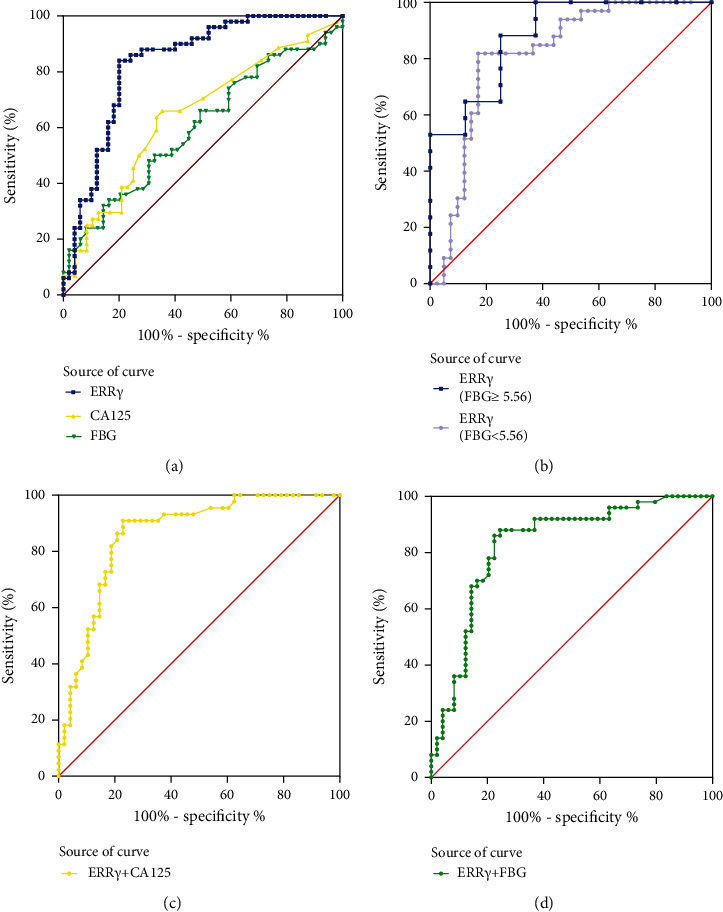
Diagnostic and prognostic value of ERR*γ* and other serological indicators for EC. (a) ROC curves of serum levels of ERR*γ*, CA125, and FBG. (b) ROC curves of serum levels of ERR*γ* under different glucose stratification. (c) The ROC curve of ERR*γ* combined with CA125. (d) The ROC curve of ERR*γ* combined with FBG. FBG: fasting blood glucose.

We further sought to evaluate the diagnostic ability of serum ERR*γ* in EC. A receiver operating characteristic (ROC) curve was used to evaluate the diagnostic value of the serum ERR*γ* level to further determine whether ERR*γ* could serve as a noninvasive biomarker (Figure 6; Table S2). The area under the ROC curve (AUC) for ERR*γ*, CA125, and FBG was 0.834, 0.648, and 0.601, respectively, in distinguishing EC patients from healthy individuals, and the ERR*γ* cutoff value was 1.050 ng/ml with a sensitivity of 84.0% and a specificity of 80.0%. Moreover, in the stratified study of patients with different FBG levels, the AUC of ERR*γ* was 0.882, and the sensitivity of ERR*γ* was increased by 4.2% in the FBG ≥5.56 mmol/L group. When ERR*γ* and other indicators were combined to diagnose EC, the AUC of ERR*γ*/CA125 was 0.861, and the predictive performance of this combination was improved (Youden index = 0.680, *P* < 0.001). These data demonstrate the potential of serum ERR*γ* as a relevant test for EC diagnosis.

### 3.8. Correlation between Serum Levels of ERR*γ* and FBG

Our results showed that serum ERR*γ* levels in subjects with FBG ≥5.56 mmol/L were significantly higher than those with FBG <5.56 mmol/L (*P*=0.006; Figure 5(c)). Then, we observed a significant positive correlation between serum ERR*γ* levels and FBG in EC patients and healthy controls, with a correlation coefficient of 0.355 (*P* < 0.001; Figure 5(d)). However, there was no significant correlation between serum ERR*γ* levels and CA125 and age (*P*=0.135, *P*=0.602; Figures 5(e) and 5(f)). These findings provided further evidence to support that the serum ERR*γ* levels were associated with FBG levels.

## 4. Discussion

In this study, we found ERR*γ* is overexpressed in both tissues and serum of EC patients. The expression level of ERR*γ* in tissues was significantly correlated with myometrial invasion in EC patients, and the level of ERR*γ* was positively correlated with the FBG level. In addition, ROC analysis showed that serum ERR*γ* has a good diagnostic performance in distinguishing EC patients from healthy people. These results suggest that ERR*γ* may be involved in regulating glucose metabolism and promoting myometrial invasion of EC and may be a noninvasive biomarker source for endometrial cancer detection and progression monitoring.

The prognostic factors of EC have been studied in detail. The most important factors include FIGO stage, myometrial invasion, histological subtypes and grades, and lymphatic invasion [2]. Among them, myometrial invasion is an important manifestation of invasion and metastasis of malignant tumor, while two recent systematic reviews and a meta-analysis showed that both deep myometrial invasion and lymphovascular space invasion have prognostic value independent of TCGA signature, as well as age and adjuvant treatment [13, 14]. ERR*γ* is one of the members of the orphan nuclear receptor [15]. With the in-depth research in recent years, it has been found that the expression of ERR*γ* is abnormal in a variety of malignant tumors and plays a role in the development of tumors [9]. In breast cancer, ERR*γ* is usually overexpressed and upregulated after acquisition of tamoxifen resistance, suggesting that ERR*γ* plays a promoting role in cancer. In prostate cancer [16], selective ERRɑ/*γ* reverse agonist SLU-PP-1072 can inhibit the Warburg effect and induce apoptosis of prostate cancer cells [17]. Sun Y et al. found that ERR*γ* was positively expressed in EC cells, and ERR*γ* could promote the proliferation of estrogen-dependent EC cells by activating the AKT-ERK1/2 signal pathway [18]. Hua T et al. reported that ERR*γ* could promote the expression of E-cadherin and participate in the migration and metastasis of EC cells [19]. The results showed that ERR*γ* was closely related to the progress of EC. In this study, bioinformatics analysis was carried out based on TCGA database, and IHC results confirmed that ERR*γ* was highly expressed in EC, and our data indicated that ERR*γ* was closely related to the deep myometrial invasion of EC, which was an invasion-related indicator with potential prognostic value. However, the prognostic relationship between ERR*γ* and different pathological and molecular types remains to be further studied.

In recent years, various clinical studies have found that EC is associated with metabolic disorders, including obesity, diabetes, and metabolic syndrome [20]. EC patients are often accompanied by systemic metabolic disorders, and hyperglycemia is the main clinical feature, which is related to poor outcome [4, 21, 22]. Our clinical data analysis found that the depth of myometrial invasion of EC was correlated with FBG, suggesting that poor blood glucose status is closely related to the development of EC. At present, more and more evidence confirms that ERR*γ* plays a central role in metabolic genes and the regulation of cellular energy metabolism [23]. Previous studies have shown that ERR*γ* can bind and regulate a variety of glycolytic gene promoters such as hexokinase 2 (Hk2), Aldolase C (Aldo-C) enolase 1, and lactate dehydrogenase A (LDHA) [24]. O-GlcNAcylation of ERR*γ* serves as a major signal to promote hepatic gluconeogenesis [25]. These results indicate that ERR*γ* is involved in maintaining glucose homeostasis in vivo, and the imbalance of glucose metabolism—high level of glycolysis—is one of the characteristics of tumor cell metabolism [26, 27]. However, there are few studies on the relationship between ERR*γ* and abnormal glucose metabolism in tumor. Our results suggest that ERR*γ* is significantly correlated with blood glucose in EC, and it is likely that ERR*γ* is involved in regulating blood glucose in EC and promoting myometrial invasion.

Current biomarkers for EC metastasis, such as immunohistochemical markers ER and PR, have great limitations and lack specificity. With the development of molecular typing of endometrial cancer, these old markers are no longer clinically useful; it is of great significance to find a new biomarker. Raffone A et al. reported that metabolomics may be suitable for a noninvasive diagnosis and screening of EC, offering the possibility to predict tumor behavior and pathological characteristics [28]. Several metabolites such as homocysteine, phospholipase-A2, and lysophospholipase-D, may be useful for diagnosis, screening, and prediction of tumor histotype, myometrial invasion, lymph vascular invasion, and cancer progression in patients with EC [28]. It is noteworthy that ERR*γ*, as a metabolism-related gene, is closely associated with tumor glucose metabolism and may be added to the list. ERR*γ* has good diagnostic performance in distinguishing EC patients from healthy people, with high sensitivity and specificity. ERR*γ* detection is not only suitable for tissue but also for serum, and with the increase of the expression level of tumor progression, it has the characteristics of tumor markers, which could have an extraordinary impact on the management of EC in the future.

Some shortcomings of this study should be acknowledged. First, the sample size in this study is relatively small, which might raise the bias of analysis. Second, it is necessary to further explore the internal mechanism of ERR*γ* regulating glucose metabolism and promoting myometrial invasion of EC. In addition, we only measured serum ERR*γ* levels in the validation phase; however, the comparative information of ERR*γ* expression in and out of cells could not be determined.

## 5. Conclusion

Collectively, ERR*γ* is overexpressed in EC and may be involved in regulating glucose metabolism and promoting myometrial invasion of EC. In addition, serum ERR*γ* has a good diagnostic performance in distinguishing EC patients from healthy people and may be a promising noninvasive biomarker in EC.

## Figures and Tables

**Table 1:** Correlation between expression of ERR*γ*, myometrial invasion depth, and immunohistochemical markers in EC.

Markers	ERR*γ* expression *n* (%)			Myometrial invasion depth *n* (%)		
	Low	High	*P* value	≥1/2	<1/2	*P* value
ER						
Negative	0 (0.0)	9 (15.3)	0.102	4 (16.6)	5 (9.1)	0.443
Positive	20 (100.0)	50 (84.7)		20 (83.3)	50 (90.9)	
PR						
Negative	0 (0.0)	10 (17.2)	0.057	5 (21.7)	5 (9.1)	0.128
Positive	20 (100.0)	48 (82.8)		18 (78.3)	50 (90.9)	
Vimentin						
Negative	11 (61.1)	13 (23.2)	0.003^*∗∗*^	3 (12.5)	30 (60.0)	0.018^*∗*^
Positive	7 (38.9)	43 (76.8)		21 (87.5)	20 (40.0)	
PTEN						
Negative	5 (35.7)	11 (52.4)	0.332	3 (33.3)	13 (50.0)	0.460
Positive	9 (64.3)	10 (47.6)		6 (66.7)	13 (50.0)	
P53						
Negative	7 (36.8)	27 (45.8)	0.495	9 (37.5)	25 (46.3)	0.470
Positive	12 (63.2)	32 (54.2)		15 (62.5)	29 (53.7)	
Ki67						
≥50%	12 (60.0)	28 (47.5)	0.332	8 (33.3)	32 (58.2)	0.042^*∗*^
<50%	8 (40.0)	31 (52.5)		16 (66.7)	23 (41.8)	

ER: estrogen; PR: progesterone receptor. ^*∗*^*P* < 0.05; ^*∗∗*^*P* < 0.01.

**Table 2:** Clinical data comparison of EC patients with different myometrial invasion depth groups.

Variable	*n*	≥1/2	*n*	<1/2	*P* value
Age (years)	141	56.96 ± 9.32	384	52.44 ± 8.08	<0.001^*∗∗*^
BMI (kg/m^2^)	112	24.56 ± 3.33	299	24.50 ± 3.46	0.875
FBG (mmol/L)	141	5.82 ± 1.59	383	5.34 ± 1.07	0.001^*∗∗*^
TG (mmol/L)	140	1.57 ± 1.14	383	1.57 ± 0.95	0.960
TC (mmol/L)	140	4.95 ± 1.09	382	4.98 ± 0.92	0.772
HDL (mmol/L)	116	1.41 ± 0.27	345	1.41 ± 0.38	0.969
LDL (mmol/L)	86	2.96 ± 0.98	259	2.87 ± 0.74	0.384
CA125 (U/mL)	139	96.90 ± 246.89	369	36.91 ± 173.12	0.009^*∗∗*^
CA15-3 (U/mL)	132	14.69 ± 20.36	362	11.07 ± 11.36	0.055
CA19-9 (U/mL)	128	207.43 ± 1020.83	325	71.40 ± 670.46	0.165
SCC (ug/L)	111	1.49 ± 1.29	290	1.25 ± 1.07	0.065
AFP (ng/mL)	130	3.47 ± 7.64	348	2.72 ± 1.41	0.268
CEA (ng/mL)	130	2.16 ± 1.46	354	2.05 ± 1.91	0.554

Notes: BMI: body mass index; FBG: fasting blood glucose; TG: triglyceride; TC: total cholesterol; HDL: high-density lipoprotein; LDL: low-density lipoprotein. ^*∗*^*P* < 0.05; ^*∗∗*^*P* < 0.01.

## Data Availability

Data used to support the findings of this study are available from the corresponding author upon request.

## Supplementary Materials

Supplementary Materials Table S1: logistic regression analysis of myometrial invasion in EC. Table S2: diagnostic value of ERR*γ*, CA125, and FBG for EC patients.

## References

[1] Siegel R. L., Miller K. D., Fuchs H. E., Jemal A. (2021). Cancer Statistics. *CA: A Cancer Journal for Clinicians*.

[2] Morice P., Leary A., Creutzberg C., Abu-Rustum N., Darai E. (2016). Endometrial cancer. *Lancet (London, England)*.

[3] Byrne F. L., Martin A. R., Kosasih M., Caruana B. T., Farrell R. (2020). The role of hyperglycemia in endometrial cancer Pathogenesis. *Cancers*.

[4] Ko E. M., Walter P., Clark L. (2014). The complex triad of obesity, diabetes and race in Type I and II endometrial cancers: prevalence and prognostic significance. *Gynecologic Oncology*.

[5] Onstad M. A., Schmandt R. E., Lu K. H. (2016). Addressing the role of obesity in endometrial cancer risk, prevention, and treatment. *Journal of Clinical Oncology*.

[6] Yoshihara E., Wei Z., Lin C. S. (2016). ERR*γ* is required for the metabolic maturation of therapeutically functional glucose-responsive *β* cells. *Cell Metabolism*.

[7] Misra J., Kim D. K., Choi H. S. (2017). ERR*γ*: a Junior orphan with a Senior role in metabolism. *Trends in Endocrinology and Metabolism*.

[8] Soundararajan A., Prabu P., Mohan V., Gibert Y., Balasubramanyam M. (2019). Novel insights of elevated systemic levels of bisphenol-A (BPA) linked to poor glycemic control, accelerated cellular senescence and insulin resistance in patients with type 2 diabetes. *Molecular and Cellular Biochemistry*.

[9] Kim J. H., Choi Y. K., Byun J. K. (2016). Estrogen-related receptor *γ* is upregulated in liver cancer and its inhibition suppresses liver cancer cell proliferation via induction of p21 and p27. *Experimental & Molecular Medicine*.

[10] Ren Z., Yang H., Wang C., Ma X. (2015). The effects of PGC-1*α* on the proliferation and energy metabolism of malignant endometrial cancer cells. *OncoTargets and Therapy*.

[11] Lu K. H., Broaddus R. R. (2020). Endometrial cancer. *New England Journal of Medicine*.

[12] Setiawan V. W., Yang H. P., Pike M. C. (2013). Type I and II endometrial cancers: have they different risk factors?. *Journal of Clinical Oncology*.

[13] Raffone A., Travaglino A., Raimondo D. (2021). Prognostic value of myometrial invasion and TCGA groups of endometrial carcinoma. *Gynecologic Oncology*.

[14] Raffone A., Travaglino A., Raimondo D. (2022). Lymphovascular space invasion in endometrial carcinoma: a prognostic factor independent from molecular signature. *Gynecologic Oncology*.

[15] Giguère V., Yang N., Segui P., Evans R. M. (1988). Identification of a new class of steroid hormone receptors. *Nature*.

[16] Madhavan S., Gusev Y., Singh S., Riggins R. B. (2015). ERR*γ* target genes are poor prognostic factors in Tamoxifen-treated breast cancer. *Journal of Experimental & Clinical Cancer Research*.

[17] Schoepke E., Billon C., Haynes K. M. (2020). A selective ERR*α*/*γ* Inverse agonist, SLU-PP-1072, inhibits the Warburg effect and induces apoptosis in prostate cancer cells. *ACS Chemical Biology*.

[18] Sun Y., Wang C., Yang H., Ma X. (2014). The effect of estrogen on the proliferation of endometrial cancer cells is mediated by ERR*γ* through AKT and ERK1/2. *European Journal of Cancer Prevention*.

[19] Hua T., Wang X., Chi S. (2018). Estrogen‑related receptor *γ* promotes the migration and metastasis of endometrial cancer cells by targeting S100A4. *Oncology Reports*.

[20] Trabert B., Wentzensen N., Felix A. S., Yang H. P., Sherman M. E., Brinton L. A. (2015). Metabolic syndrome and risk of endometrial cancer in the United States: a study in the SEER-medicare linked database. *Cancer Epidemiology Biomarkers & Prevention*.

[21] Ni J., Zhu T., Zhao L. (2015). Metabolic syndrome is an independent prognostic factor for endometrial adenocarcinoma. *Clinical and Translational Oncology*.

[22] Nagle C. M., Crosbie E. J., Brand A. (2018). The association between diabetes, comorbidities, body mass index and all-cause and cause-specific mortality among women with endometrial cancer. *Gynecologic Oncology*.

[23] Kida Y. S., Kawamura T., Wei Z. (2015). ERRs mediate a metabolic Switch Required for Somatic cell Reprogramming to Pluripotency. *Cell Stem Cell*.

[24] Cai Q., Lin T., Kamarajugadda S., Lu J. (2013). Regulation of glycolysis and the Warburg effect by estrogen-related receptors. *Oncogene*.

[25] Misra J., Kim D.-K., Jung Y. S. (2016). O-GlcNAcylation of orphan nuclear receptor estrogen-related receptor *γ* promotes hepatic gluconeogenesis. *Diabetes*.

[26] Asgari Y., Zabihinpour Z., Salehzadeh-Yazdi A., Schreiber F., Masoudi-Nejad A. (2015). Alterations in cancer cell metabolism: the Warburg effect and metabolic adaptation. *Genomics*.

[27] Hsu P. P., Sabatini D. M. (2008). Cancer cell metabolism: Warburg and beyond. *Cell*.

[28] Raffone A., Troisi J., Boccia D. (2020). Metabolomics in endometrial cancer diagnosis: a systematic review. *Acta Obstetricia et Gynecologica Scandinavica*.

