# Supplementary material for: ERRγ, a Novel Biomarker, Associates with Pathoglycemia of Endometrial Cancer to Predict Myometrial Invasion
Source: J Oncol. 2022 Jun 21;2022:5283388. doi: 10.1155/2022/5283388 (PMC9239760; doi:10.1155/2022/5283388)
Supplement: Supplementary Materials — Table S1: logistic regression analysis of myometrial invasion in EC. Table S2: diagnostic value of ERRγ, CA125, and FBG for EC patients. [file 5283388.f1.docx]

Table S1 Logistic regression analysis of myometrial invasion in EC

| Factors | β | SE | P | OR | 95%Cl |
| --- | --- | --- | --- | --- | --- |
| FBG | 0.248 | 0.077 | 0.001** | 1.281 | 1.102-1.490 |
| CA125 | 0.002 | 0.001 | 0.019* | 1.002 | 1.000-1.004 |

Logistic regression analysis was used after adjusting for age to identify the possible risk factors for myometrial invasion. SE: standard error, OR: odds ratio, CI: confidence interval, FBG: fasting blood glucose. *P˂0.05, **P˂0.01.

Table S2 Diagnostic value of ERRγ, CA125 and FBG for EC patients

| Variables | n | AUC | Cut off | Sensitivity | Specificity | Youden index | 95% confidence interval | |  |
| --- | --- | --- | --- | --- | --- | --- | --- | --- | --- |
|  |  |  |  |  |  |  | Lower limit | Upper limit | P-Value |
| FBG | 99 | 0.601 | 5.56 | 0.340 | 0.837 | 0.177 | 0.489 | 0.713 | 0.084 |
| CA125 | 92 | 0.648 | 15.050 | 0.659 | 0.667 | 0.326 | 0.535 | 0.761 | 0.015* |
| ERRγ | 100 | 0.834 | 1.050 | 0.840 | 0.800 | 0.640 | 0.753 | 0.916 | ˂0.001** |
| FBG≥ 5.56 | 25 | 0.882 | 1.153 | 0.882 | 0.750 | 0.632 | 0.738 | 1.000 | 0.002** |
| FBG˂ 5.56 | 74 | 0.815 | 1.049 | 0.818 | 0.829 | 0.647 | 0.714 | 0.916 | ˂0.001** |
| ERRγ+FBG | 99 | 0.829 | - | 0.860 | 0.776 | 0.636 | 0.744 | 0.914 | ˂0.001** |
| ERRγ+CA125 | 92 | 0.861 | - | 0.909 | 0.771 | 0.680 | 0.784 | 0.938 | ˂0.001** |

Notes:FBG: fasting blood glucose. *P˂0.05, **P˂0.01.
